# Dynamic reconfiguration and transition of whole-brain networks in patients with MELAS revealed by a hidden Markov model

**DOI:** 10.3389/fneur.2025.1625888

**Published:** 2025-09-22

**Authors:** Qingyun Yu, Rong Wang, Chong Sun, Bin Hu, Xueling Liu, Liqin Yang, Jie Lin, Daoying Geng, Yuxin Li

**Affiliations:** ^1^Shanghai Institute of Infectious Disease and Biosecurity, Fudan University, Shanghai, China; ^2^Department of Radiology, HuaShan Hospital, Fudan University, Shanghai, China; ^3^Department of Neurology, HuaShan Hospital, Fudan University, Shanghai, China; ^4^Shanghai Engineering Research Center of Intelligent Imaging for Critical Brain Diseases, Shanghai, China

**Keywords:** MELAS, stroke-like episode, rs-fMRI, hidden Markov model, whole-brain network dynamics

## Abstract

**Objectives:**

Mitochondrial encephalomyopathy with lactic acidosis and stroke-like episodes (MELAS) is a rare maternally inherited disease. The neuropathologic mechanisms and neural network alterations underlying stroke-like episodes (SLEs), a recurrent paroxysmal clinical event, remain unclear. The hidden Markov model (HMM) can detect profound alterations in neural activities across the whole-brain network.

**Materials and methods:**

We initially collected data from a prospective cohort from 2019 to 2024. The confirmed diagnosis of MELAS was conducted through genetic testing or a muscle biopsy. Healthy control volunteers were recruited from the local community. By utilizing the HMM, we evaluated the temporal characteristics and transitions of HMM states and the specific community pattern of transitions and activation maps of the whole brain for subjects.

**Results:**

Thirty-six MELAS patients at the acute stage (MELAS-acute group) and 30 healthy controls (HC group) were included in this study. Based on HMM, fractional occupancies in states 5 and 6 for MELAS were significantly decreased (*p* < 0.001), but fractional occupancies in states 2, 3, 4, 7, 8, 9, 10, and 11 were significantly increased (*p* < 0.05), compared to HCs. The lifetimes of HMM states showed a similar decrease as fractional occupancies. The switching frequency of HMM states was significantly increased in MELAS (*p* < 0.001). Combined with the special community patterns of transitions, MELAS displayed differential activity patterns in crucial areas of the default mode network (DMN) and visual network (VN).

**Conclusion:**

This study suggests dynamic reconfiguration of HMM states, special transition modules, and multiple transition pathways in MELAS, providing novel insights into the neural network mechanisms underlying MELAS.

## Introduction

Mitochondrial encephalomyopathy with lactic acidosis and stroke-like episodes (MELAS) is a rare maternally inherited disease caused by mutations in mitochondrial or nuclear genes (1). Up to 80% of MELAS patients carry the m.3243A > G mutation in the MT-TL1 gene encoding tRNA^Leu(UUR)^ (2). Especially, a stroke-like episode (SLE) is a recurrent paroxysmal clinical event that usually presents seizures, headaches, cortical blindness, motor weakness, and cognitive impairment (1, 3). SLEs associated with some signal abnormalities resolve completely; however, most lesions develop into cortical laminar necrosis, gliosis, and atrophy, resulting in the gradual accumulation of neurological dysfunction, which is heterogeneous (3). Previous studies have shown that SLEs were primarily seizure-induced but not driven by angiopathic changes in the mechanism (4). Despite significant research efforts, the precise neuropathological mechanisms and neural network alterations underlying the progression of SLEs in MELAS remain unclear, highlighting the complexities of this disease.

Resting-state functional magnetic resonance imaging (rs-fMRI) can quantify intrinsic functional brain organization and measure synchronizations between spontaneous events in non-adjacent brain regions (5). Wang et al. (6) investigated dynamic functional connectivity (dFC) in MELAS using a sliding window approach (SWA) to characterize global connectivity patterns, and the results showed that MELAS patients exhibited an inability to switch out of a state with weak connectivity into more highly and specifically connected network configurations, which was more significant at the acute stage of SLEs. This finding strongly suggested that MELAS patients, particularly in the acute phase, may experience disruptions in their brain network dynamics, leading to atypical patterns of connectivity transitions. Given that alterations in brain energy metabolism and neuronal integrity are central to MELAS pathophysiology, it might be plausible that the underlying damage affects the timely and efficient re-establishment of brain functional networks. However, this approach still has some limitations. Covariance matrices based on different windows are classified into different states by clustering analysis, which is highly dependent on the predetermination of time scales and neglects temporal variances of network fluctuations (7). Given the paroxysmal and recurrent nature of SLEs in MELAS, the underlying mitochondrial dysfunction could lead to fluctuating neuronal excitability and impaired energy metabolism, potentially causing transient disruptions in brain network connectivity. While the SWA offers a time-varying perspective, its reliance on pre-determined window lengths may not optimally capture the complex, rapidly changing, and quasi-stationary network states that could arise from such pathological processes.

On the other hand, the hidden Markov model (HMM), as a generative probabilistic model, is capable of characterizing the dynamics of brain activity by capturing distinct spatial patterns across the whole brain, which are inferred directly from the dataset without the need for sliding window predetermination (8, 9). The HMM assumes that time-series data can be described using a hidden sequence of a finite number of states, such that, at each time point, only one state is active (10). All states have the same probabilistic distributions, but each has different distribution parameters. Hence, HMM states correspond to unique patterns of brain activity that recur in different parts of the time series (8). Then, we can capture the temporal characteristics of each state from time courses. Previous studies have demonstrated that the HMM can identify dynamic reorganization of whole-brain networks on minimal time scales, especially, the HMM can capture quasi-stationary states of activity that are consistently recurring over the population (9, 11, 12). Therefore, the HMM’s ability to capture quasi-stationary states offers a more dynamic and flexible framework to characterize the altered whole-brain networks in MELAS, potentially reflecting the brain’s struggle to maintain stable network configurations due to metabolic deficits and neuronal damage. To quantify these dynamic network alterations, we would analyze key temporal characteristics derived from the HMM, fractional occupancy (FO), representing the proportion of time spent in each state; lifetime (LT), indicating the average duration spent in a given state; and transition probability (TP), describing the rate of switching between states (8). In the context of SLEs, abnormalities in these metrics were hypothesized to reflect the impact of mitochondrial dysfunction on brain network stability and efficiency.

Based on these considerations, we hypothesized that MELAS-acute patients would exhibit abnormal neural network dynamics, characterized by atypical patterns of transitions across distinct brain network states. Furthermore, we proposed that these altered spatiotemporal characteristics, as quantified by FO, LT, and TP, could be associated with the physical and cognitive performance of MELAS patients. To test this hypothesis, this study inferred the HMM states and dynamic reconfiguration of whole-brain networks based on the rs-fMRI data of MELAS patients and healthy controls. Specifically, we evaluated the differences in the spatiotemporal characteristics of HMM states and special community patterns of transitions between these groups. In addition, we explored the possible correlations between these temporal metrics of HMM states and clinically relevant features of MELAS patients.

## Materials and methods

### Study participants

This study was approved by the Institutional Review Board of Huashan Hospital and followed the tenets of the Declaration of Helsinki. Written informed consent was obtained from all participants before inclusion.

We initially collected a prospective cohort from 2019 to 2024. The present study undertook a cross-sectional analysis based on data extracted from the prospective cohort study, utilizing information from a single observation point. All the MELAS patients included were diagnosed by a specialized expert (J. L.) who had more than 15 years’ experience in mitochondrial myopathy. The diagnosis of MELAS was confirmed by genetic testing or muscle biopsy (1). Specifically, in this study, we only recruited the MELAS patients carrying the m.3243A > G mutation. Forty-four MELAS patients at the acute stage (MELAS-acute group, within 1 week after SLE) were enrolled from June 2019 to August 2024, and we have collected demographic and physical data, mainly including age, gender, frequency of SLE, the symptoms during SLE, serum lactate, creatine kinase (CK), and lactate dehydrogenase (LDH). Moreover, 30 healthy control volunteers (HC group) who were recruited from the local community participated, were gender- and age-matched. All the subjects were right-handed.

The exclusion criteria included the following: (1) participants with psychiatric or neurodegenerative diseases (such as autism, major depressive disorder, bipolar disorder, and Parkinson’s disease); (2) those presenting other organic brain lesions; (3) head trauma; (4) history of drug abuse or alcohol addiction; and (5) those with an inability to complete the MRI examination.

### MRI data acquisition

MRI data were acquired using a 3.0 T GE scanner with an 8-channel head coil (Discovery MR750, General Electric, Boston, MA). During scanning, all participants were commanded to keep their eyes closed, but not to fall asleep or think about anything. A single-shot gradient-recalled echo planar imaging (EPI) sequence was used to obtain the rs-fMRI data with the following parameters: echo time (TE) = 30 ms, repetition time (TR) = 2,000 ms, flip angle = 90°, slices = 35, slice thickness = 4 mm, matrix size = 64 × 64, field of view (FOV) = 240 × 240 mm, number of volumes = 210. High-resolution 3D T1-weighted images were obtained by a brain volume (BRAVO) sequence: TE = 3.2 ms, TR = 8.2 ms, flip angle = 12°, slices = 170, slice thickness = 1.2 mm, matrix size = 256 × 256, FOV = 240 mm × 240 mm.

### Rs-fMRI data preprocessing

The rs-fMRI data were preprocessed by applying the DPABI toolbox^1^ (13) and statistical parametric mapping (SPM12)^2^ (14). Specifically, we removed the first 10 image volumes, implemented slice-timing, corrected head motion, and calculated the mean frame-wise displacement (FD) (15). Subjects were excluded who had a head motion >3 mm or a 3° rotation or a mean FD of >0.25 mm (eight MELAS patients). The final sample included 66 subjects (36 in the MELAS-acute group and 30 in the HC group). Then, the rs-fMRI data were spatially normalized to the Montreal Neurological Institute (MNI) space by applying the DARTEL algorithm and were resampled to a voxel size of 3 × 3 × 3 mm^3^ (16). Next, the normalized data were spatially smoothed with a 6-mm full-width using a Gaussian kernel. Then, nuisance signals were regressed out of each voxel’s time course, including 24-parameter head-motion profiles, mean white matter (WM), cerebrospinal fluid (CSF) time series, and global signal within the respective brain masks derived from prior probability maps in SPM12 (17). Finally, the resulting images were further temporally band-pass filtered (0.01–0.1 Hz) to reduce the effects of low-frequency drift and high-frequency physiological noise (18).

### Hidden Markov model

**Figure 1:**
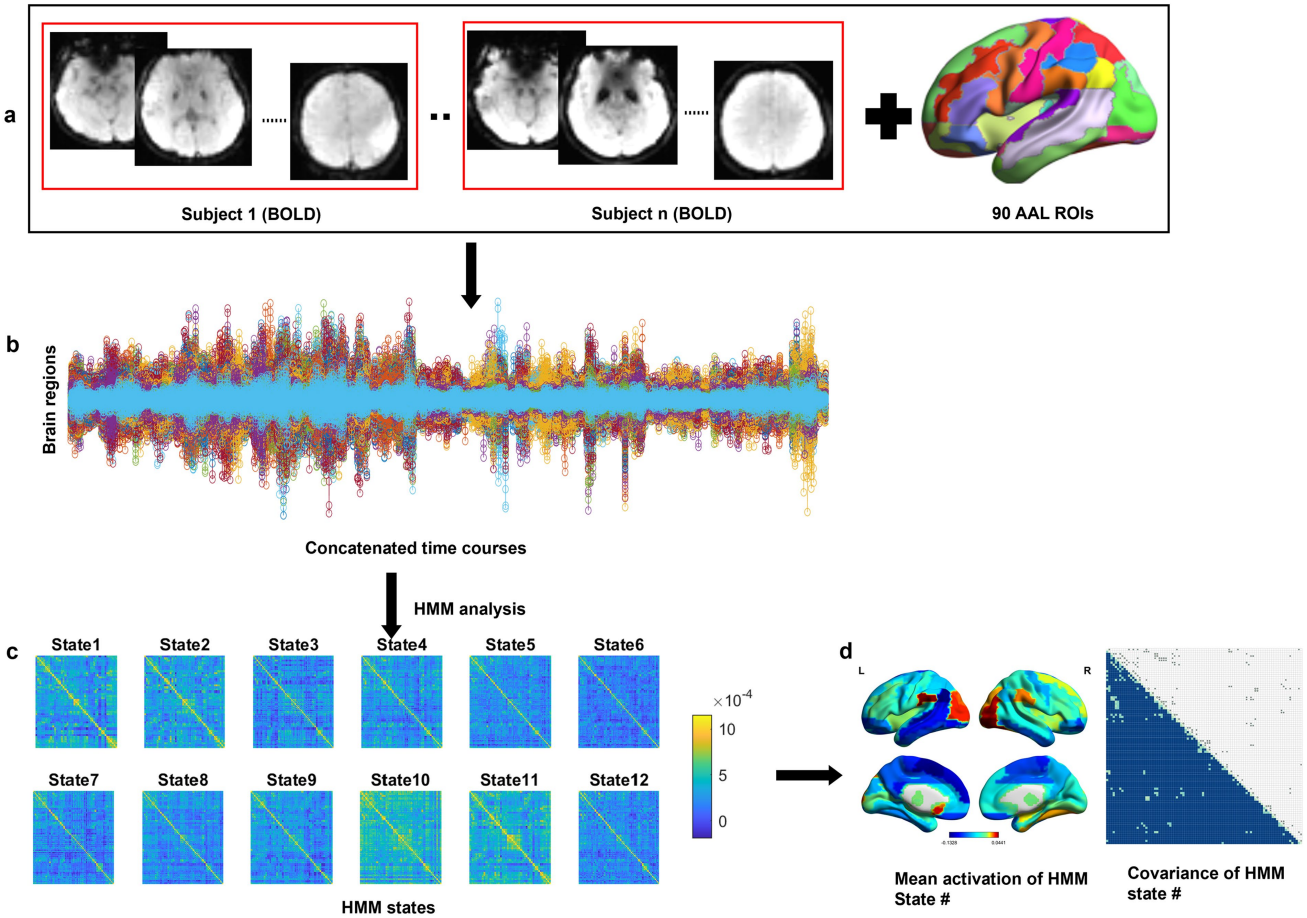
Schematic workflow with an HMM. **(a)** Whole-brain regions were parcellated into 90 ROIs based on the AAL atlas, and the ROI time courses were extracted by averaging the BOLD signal within each ROI for each participant. **(b)** The data were concatenated across all participants, including 90 brain regions × 200 time points for each subject. **(c)** The HMM analysis was run on the time courses, and 12 HMM states were obtained. **(d)** Each HMM state was characterized as a multivariate Gaussian distribution, including a covariance matrix and a mean activation. Abbreviations: AAL, automated anatomical labeling; BOLD, blood oxygen level-dependent imaging; HMM, hidden Markov model; ROI, regions of interest.

The HMM was implemented using variational Bayesian inference to probabilistically estimate the state statistics and transition probabilities (10). To explore the dynamics of whole-brain networks, we applied the HMM to time courses extracted from cerebral regions. In the most common variant of the HMM, each state is featured by a multivariate Gaussian distribution, which includes a mean activation and a covariance matrix (7, 8). During HMM inference, a central and free parameter is the number of states K, which has to be chosen before further evaluation (9, 10). Although there were some approaches to guide the choice of the number of states (8–10), such as using quantitative measures such as free energy or using non-parametric approaches, in practice, different numbers of states offer only different levels of detail of brain dynamics. The free energy is the statistical measure that is minimized during the variational inference Bayesian optimization process (10). In general, it is an approximation to the model evidence, including how well the model fits the data and the complexity of the model (10). Thus, free energy is a reasonable criterion for choosing the suitable number of states for the HMM. In addition, the median fractional occupancy is used to help determine the optimal number of states K in the HMM model. A low and stable median fractional occupancy across states, with little improvement beyond a certain K value, indicates that adding more states does not significantly improve the model’s ability to capture distinct dynamic patterns, thus influencing the selection of the appropriate K value (8). Hence, by utilizing the minimum free energy and medial fractional occupancy, we explored the best choice on state K where the similarity was minimized among different mean activations, according to previous studies (8–10). The steps of the main HMM analysis are as follows: First, according to the automated anatomical labeling (AAL), cerebral regions of each subject were segmented into 90 regions of interest (ROIs) (Figure 1a) (19). Next, the featured time courses were extracted. A total of 90 ROI time courses across all participants were temporally concatenated, producing a single concatenated course from the inferred 90*(66*200) with 200 time points (Figure 1b). Furthermore, the HMM analysis was run on time courses, and 12 HMM states were obtained (Figure 1c). Finally, each recurring state was featured in a mean activation and covariance matrix (Figure 1d).

### Decoding of HMM states

We applied Neurosynth to decode the potential functions of HMM states (20). We submitted mean activation maps of HMM states to the Neurosynth. In descending order, we ranked all correlation coefficients and showed approximately 10–15 terms of maximum correlation for each state.

### Analysis of dynamic temporal characteristics and transitions in HMM states

We defined fractional occupancies (FO), lifetimes (LT), and switching frequency (SF) to depict the temporal characteristics of HMM states, which are referred to as the temporal proportions in a state, time spent in a state before transferring into another state, and the frequency of transitions between different states, respectively (8). Global activity dynamics of HMM states were evaluated from the time course of posterior probabilities (8, 12).

After obtaining these states, we calculated the transition probability (TP) matrix for each participant. Furthermore, we used a community detection approach to elucidate common HMM states in the TP matrix, which indicate more frequent transitions within states. Especially, using a common community detection approach, the Louvain-like locally greedy heuristic algorithm, we applied modularity maximization to choose the following modularity quality function (21, 22). According to the previous study, we thresholded the transition matrix before running the modularity algorithm, which included 21% of the strongest transitions (23).

### Statistical analysis

A two-tailed two-sample *t*-test was applied to analyze group differences in age between MELAS and HCs. A Mann–Whitney U-test was used to analyze differences in dynamic temporal characteristics of HMM states for each scan site, and the effect size was quantified using the rank biserial correlation (r). A chi-square test was applied to analyze gender-ratio differences between the groups. We conducted a permutation test to demonstrate the TP of HMM states between the groups. By running 5,000 permutations across participants, we established a null distribution for global dynamic differences between each state and between groups, producing *p*-values. In addition, the non-parametric Spearman’s correlation coefficient was calculated to evaluate the correlations between the temporal characteristics of HMM states and clinical-related features of MELAS patients.

Statistical analysis was executed using GraphPad Prism version 10.2.3 for Windows (GraphPad Software, San Diego, California, United States),^3^ and MATLAB software version R2023b [The MathWorks, Inc. (Year). MATLAB (Version R2023b)].^4^ The significance level was set to a *p*-value of <0.05 after false discovery rate (FDR) correction.

## Results

### Demographic and clinical features

The demographic and clinical features are presented in Table 1. There were no significant group differences in age (*p* = 0.192) or gender (*p* = 0.498). The mean age of the first stroke-like episode was 24 years old, and the median time interval of a second stroke-like episode was 11 months. MELAS-acute patients manifested SLE symptoms, usually including seizure, headache, cortical blindness, motor weakness, cognitive impairment, vomiting, hearing loss, and aphasia. Serum lactate, CK, and LDH data were available for analysis in 28, 30, and 30 patients, respectively (*n* = 44; 64, 68, 68%).

**Table 1:** Demographic and clinical characteristics of MELAS-acute patients and HCs.

Characteristics	MELAS-acute (*n* = 36)	HC (*n* = 30)	*p*-value
Age (y, SD)	25.0 ± 9.4	28.5 ± 6.3	0.192 ^a^
Gender (M/F)	21/15	15/15	0.498 ^b^
Mean age of first stroke-like episode (y, SD)	24.0 ± 10.0	–	–
Median time interval of a second stroke-like episode (m)	11.0[3.5, 25.3]	–	–
Median frequency of SLE			
In 1 year	1[1,2]	–	–
In 2 years	2[1,3]	–	–
In 5 years	3[2,4]	–	–
Mean content of serum lactate(mmol/L)	2.4 ± 1.43	–	–
Mean content of creatine kinase(U/L)	301 ± 363	–	–
Mean content of LDH(U/L)	250 ± 128	–	–
SLE symptoms (n, %)			
Seizure	26 (72.2%)	–	–
Headache	19 (52.8%)	–	–
Cortical blindness	19 (52.8%)	–	–
Motor weakness	15 (41.7%)	–	–
Cognitive impairment	15(41.7%)	–	–
Vomiting	9 (25%)	–	–
Hearing loss	9 (25%)	–	–
Aphasia	7 (19.4%)	–	–

For continuous variables, data are expressed as the mean ± standard deviation; numbers for gender data; for count variables, data are expressed as the median and interquartile range. ^a^The *p*-value for age was obtained by a two-tailed two-sample *t*-test. ^b^The *p*-value for gender distribution was obtained by the chi-square test. MELAS-acute, MELAS patients at the acute stage; HC, healthy control; M, male; F, female; LDH: lactate dehydrogenase; SLE, stroke-like episode.

### States inferred by the HMM

**Figure 2:**
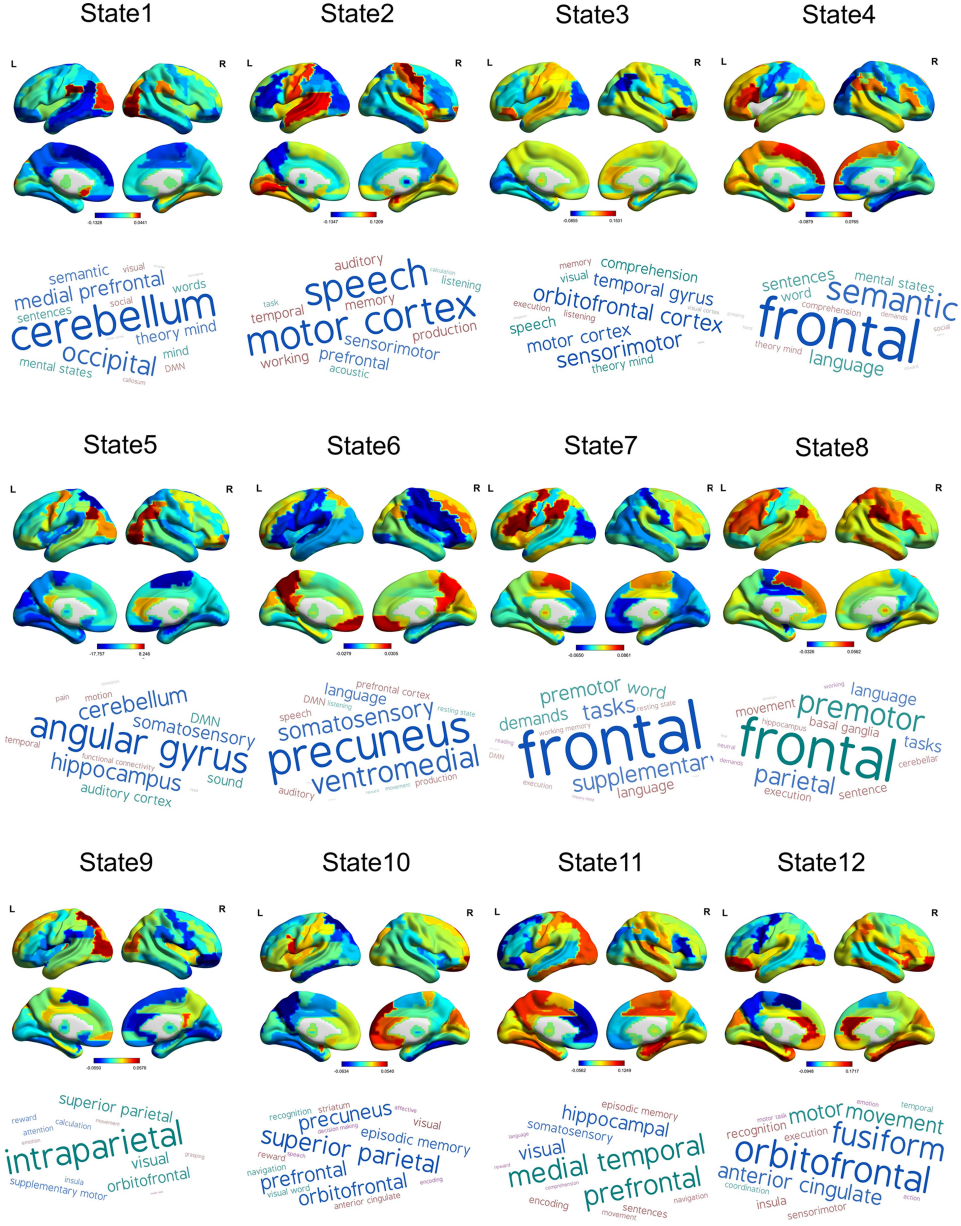
States inferred by HMM. Mean activation maps for 12 inferred states (the first row) and potential psychological function decoded by Neurosynth (the second row).

To identify the optimal number of state K for the HMM, we ran the HMM for model orders spanning 4–20 and evaluated each solution by minimum free energy and median fractional occupancy across the HMM states (Supplementary Figure S2 in supplementary materials). Generally, as K increased, the free energy initially decreased, indicating improved model fit due to greater flexibility. However, increasing K beyond a certain point resulted in an increase in free energy, signifying overfitting and excessive model complexity (Supplementary Figure S2A). The optimal states K typically correspond to the “elbow” or inflection point of the free energy curve, where the rate of improvement sharply changes (8). In this study, *K* = 12 represented this point of diminishing returns, balancing model accuracy and complexity effectively. The median fractional occupancy across states remained relatively low and stable without substantial improvement beyond *K* = 12, implying that additional states do not contribute meaningfully to capturing distinct dynamic patterns (Supplementary Figure S2B). In summary, *K* = 12 was chosen as it minimized the free energy, mitigated overfitting risks, and provided states with reasonable fractional occupancy, ensuring both statistical robustness and interpretability, and each of which was defined as a mean activation (Figure 2, the first row) and a covariance matrix (Supplementary Figure S1). In addition, we further probed the potential neuropsychological functions of HMM states by utilizing the Neurosynth (Figure 2, the second row). The results showed that state 1 was related to cognitive and visual terms; state 2 was associated with motion and speech terms; states 3 and 12 were mainly overlapped with executive, sensorimotor, and visual terms; state 4 was related to emotional and somatosensory terms; state 5 corresponded to default mode network (DMN) terms; states 6 and state 9 were mainly associated with visual and somatosensory terms; state 7 was related to executive function and emotion; state 8 was associated with movement and executive function; and states 10 and 11 were associated with sensorimotor, memory, and visual terms.

### Alterations in temporal characteristics and transition patterns for MELAS

**Figure 3:**
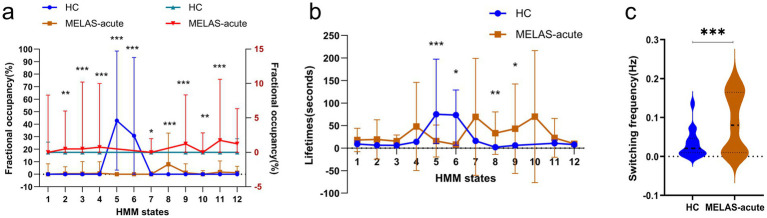
Dynamic alteration in the whole-brain network between the MELAS-acute group and the HC group. **(a)** The alterations in fractional occupancies (FO) of each state. **(b)** The alterations in lifetimes (LT) of each state. **(c)** The alterations in switching frequency (SF) of each state. The error bar represents the standard error of the median. Thick lines and thin lines represent median and interquartile range (IQR), respectively. Colors distinguished groups. For FO < 10%, the right-hand y-axis provided an expanded scale, while the left-hand y-axis pertained to all values. All temporal properties were evaluated using a Mann–Whitney U-test and the permutation test (TP), FDR corrected. *Significant differences between the different groups of the MELAS-acute group and the HC group. *p < 0.05; **p < 0.01; ***p < 0.001.

Compared to the HC group, FO of states 5 and 6 for the MELAS-acute group were significantly decreased (state 5: *p* < 0.001, *r* = −0.68; state 6: *p* < 0.001,*r* = −0.50; FDR corrected), while FO of states 2, 3, 4, 7, 8, 9, 10, and 11 was increased (state 2: *p* = 0.003, *r* = −0.42; state 3: *p* <0.001, *r* = −0.44; state 4: *p* < 0.001, *r* = −0.44; state 7: *p* = 0.014, *r* = −0.33; state 8: *p* < 0.001, *r* = −0.54; state 9: *p* <0.001, *r* = −0.50; state 10: *p* = 0.005, *r* = −0.38; state 11: *p* < 0.001, *r* = −0.52; FDR corrected) (Figure 3a). The HMM states 8 and 9 had longer LTs for MELAS-acute group (state 8: *p* = 0.007, *r* = −0.85; state 9: *p* = 0.045, *r* = −0.85; FDR corrected), and HMM state 5 and 6 had shorter LTs (state 5: *p* < 0.001, *r* = −0.80; state 6: *p* = 0.019, *r* = −0.80; FDR corrected) (Figure 3b). These findings demonstrated that MELAS patients showed specific reorganization of brain microstates.

We further compared the TP of HMM states between the groups by applying permutation analysis. Compared to the HC group, the switching frequency of HMM states for the MELAS-acute group was significantly increased (*p* < 0.001) (Figure 3c), which could elucidate that there are more unstable dynamic network patterns and transition communication between brain networks in MELAS-acute patients. However, we found no significant differences in TP between HMM states.

### The specific community pattern of transitions and activation map of HMM states for MELAS

**Figure 4:**
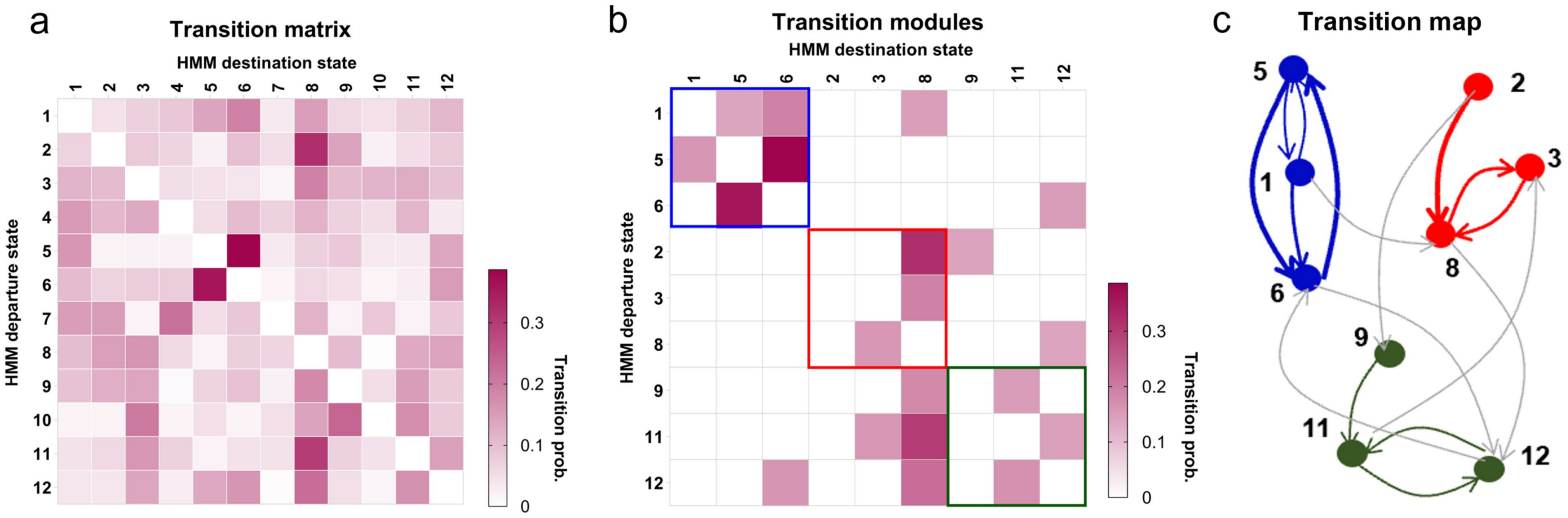
Modules of transitions between states for the MELAS-acute group and the HC group. **(a)** The probability matrix of the 12 HMM states across all participants. Each matrix entry represented the transition probability from departure state to destination state. **(b)** Three modules of the 21% strongest transitions of the HMM states. **(c)** The transition map for each HMM state. Arrows showed the direction of the transitions with thickness proportional to the transition probability.

To investigate the transition patterns of HMM states, we extracted HMM states that transition more often between each other (Figure 4a). Specifically, we applied a threshold to the state transition matrix to retain only the strongest transitions (representing 21% of the total transitions). Our analysis revealed that states, corresponding to indices 4, 7, and 10 in the initial set of states, fell into this category of low participant-wise occurrence. Therefore, the model presented with nine states reflected robust states that consistently emerged across the participant sample after applying our stringent inclusion criteria. Combined with the community patterns and global transitions (Figure 4b), we identified three modules: the MELAS-related module (red), the HC-related module (blue), and an intermediate or shared module (green), which appeared to encompass states with patterns of community organization presented in both MELAS and HC participants (Figure 4c). Specifically, states in the MELAS-related module exhibited higher FOs, longer LTs, and greater global temporal characteristics compared to those in the HC-related module. The reorganization of transition patterns indicates a special whole-brain network module associated with MELAS-acute patients. The third module (green) likely represented a shared or transitional network configuration observed across both groups. We hypothesized that this meta-state might reflect common underlying neural processes or flexible connectivity patterns not specific to either the MELAS or the HC group. Unfortunately, the clinical or functional significance of this shared module remains unclear at present.

**Figure 5:**
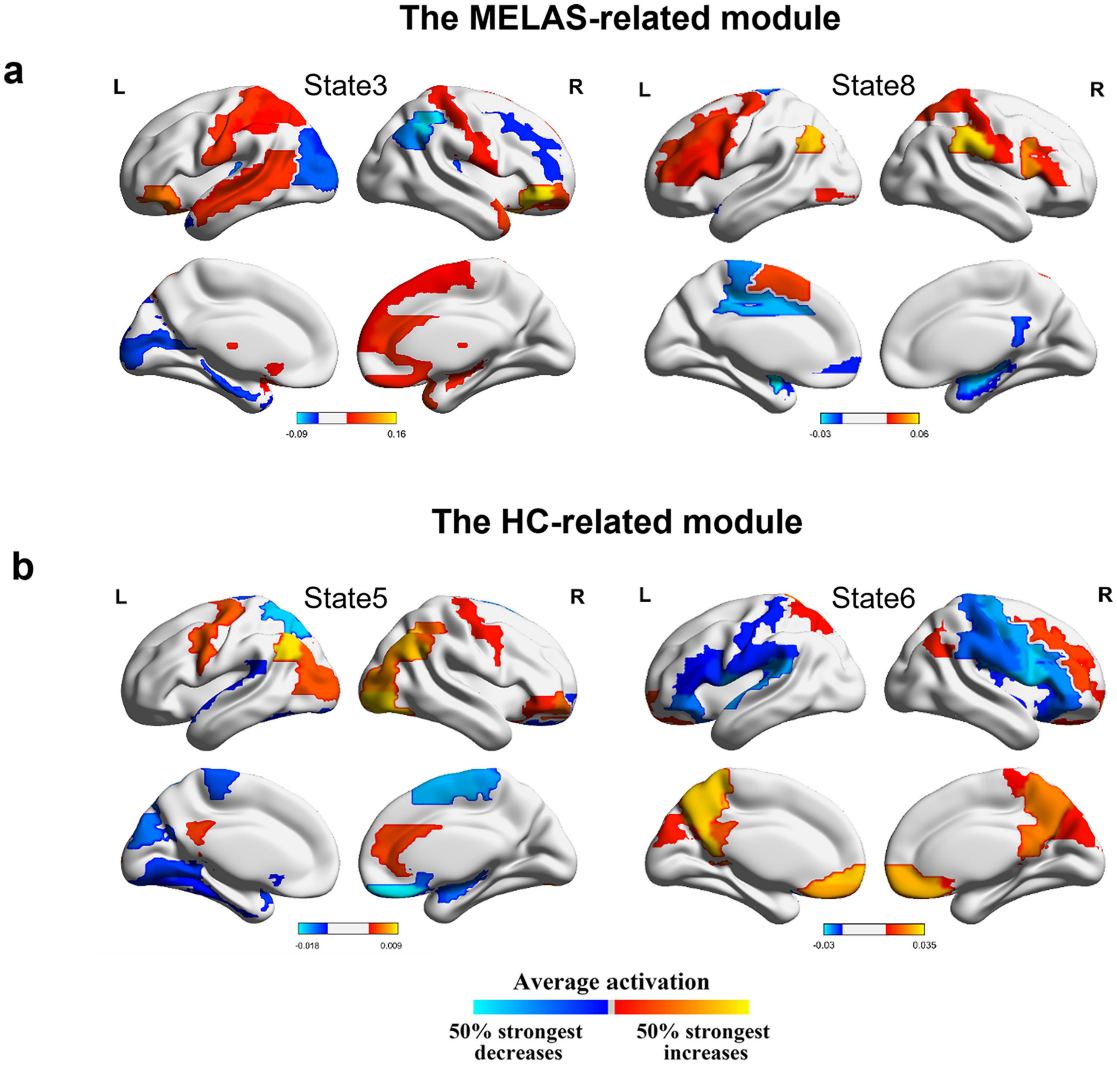
**(a)** Mean activation distribution of the MELAS-related HMM states. **(b)** Mean activation distribution of the HC-related HMM states. All maps were thresholded, respectively, above 50% strongest positive and below the negative changes.

We further investigated activation maps of the whole-brain network of different modules. The MELAS-related module was primarily featured in states 3 and 8 (Figure 5a). Combined with Neurosynth decoding of the key activated brain regions for each HMM state, we observed that MELAS-related modules exhibited distinct activity alterations in the DMN- and visual network (VN)-related brain areas. The areas are crucial for cognitive processing and sensory integration. State 3, characterized by terms related to executive function, sensorimotor engagement, and visual processing, showed a complex pattern of decreased activity in insula, sensorimotor, visual, and DMN regions, alongside increased activity in sensorimotor, visual, and auditory areas. This suggested a chaotic and inefficient network state where processing is fragmented and possibly hyperactive in some sensory-motor loops, failing to integrate information effectively, which could underlie weakness and impaired visual processing. State 8, predominantly linked to movement and executive function, presented decreased activity in DMN, visual, and sensorimotor areas. The reduced engagement of these networks during this state might signify diminished cognitive reserve or impaired sensorimotor integration, contributing to the cognitive and motor deficits observed in MELAS. On the other hand, the HC-related module is primarily characterized by states 5 and 6 (Figure 5b). HMM state 5 revealed decreased activities in sensorimotor and visual areas, alongside increased activities in DMN areas. HMM state 6 displayed decreased activities in sensorimotor areas and bilateral insula, while increased activities were observed in sensorimotor and DMN areas. Combined with the special community pattern, we found that the MELAS-acute group exhibited distinct activity alterations primarily in the crucial areas of DMN and VN compared to the HC group.

### Correlation between temporal characteristics of HMM states and clinical-related features in MELAS patients

**Figure 6:**
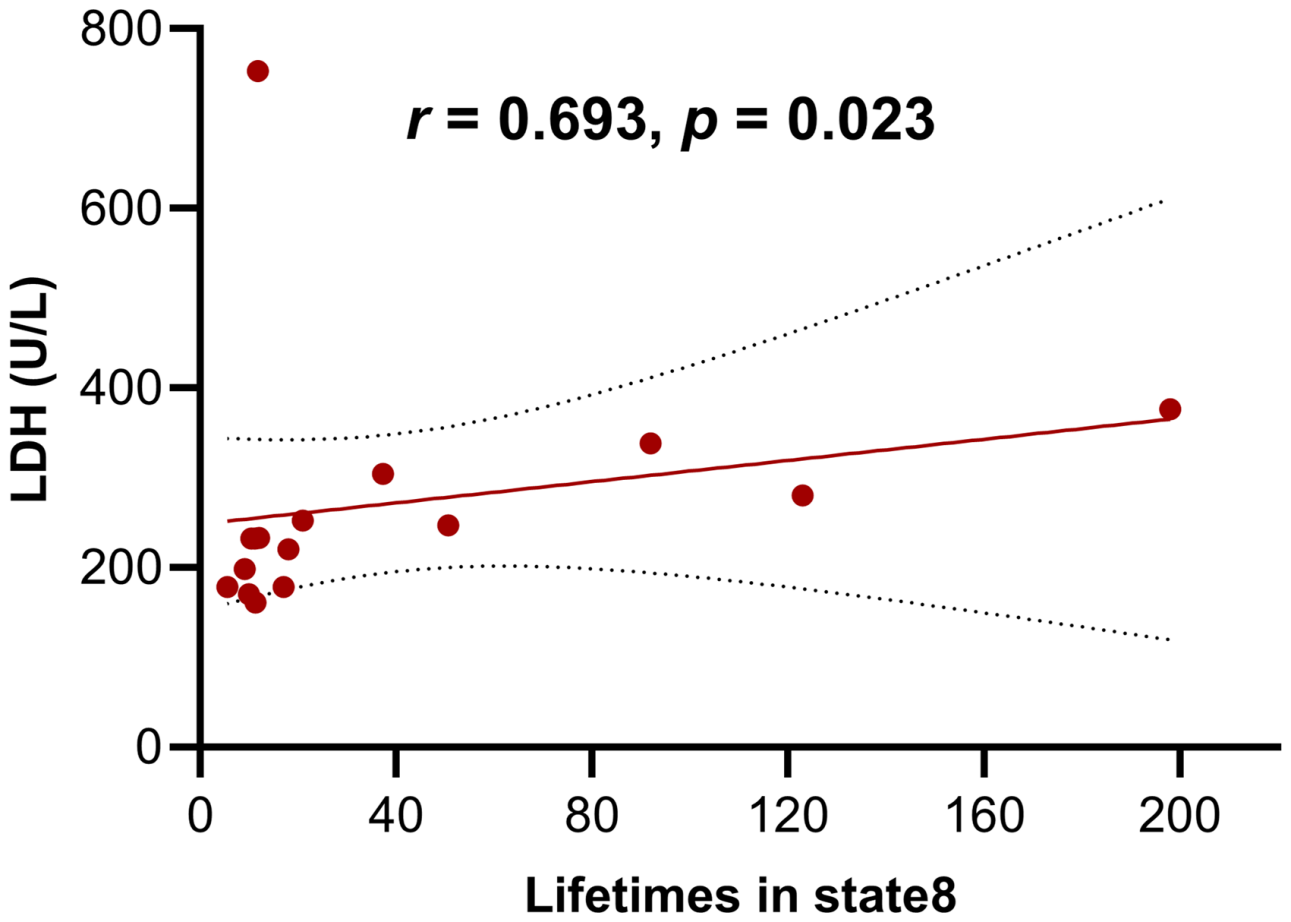
Correlations between the temporal characteristics of HMM states and clinical-related features in MELAS patients.

As shown in Figure 6, in MELAS-acute patients, the concentration of LDH was positively correlated with lifetimes in state 8 (*r* = 0.693, *p* < 0.05; FDR-corrected). There was no significant correlation between the temporal properties of other HMM states and clinically related characteristics, such as age of first SLE, time interval of a second SLE, frequency of SLE, concentration of serum lactate, and CK.

## Discussion

By using HMM inference analysis, this study demonstrated 12 HMM states characterized by unique spatiotemporal patterns of the whole brain in MELAS and HCs. Importantly, MELAS patients exhibited altered dynamic reconfiguration of specific network modules, mainly in the crucial areas of the DMN and VN. Moreover, we discovered that the concentration of LDH was correlated with the lifetimes in state 8.

Resting-state brain activity has been widely used to analyze large-scale brain networks, and dynamic alterations of whole-brain networks are essential to the knowledge of neural network mechanisms in neuropsychiatric diseases. HMM has successfully been used to investigate the dynamic reconfiguration of whole-brain networks (7). However, during HMM inference, a free and crucial parameter is the number of states, which must be chosen before further evaluation (9, 10). Although there were some approaches to guide the choice of the number of states (8–11), different numbers of states offer varying levels of detail in brain activity dynamics, which is consistent with previous studies suggesting that FC in the whole brain is highly dynamic and flexible for functional coordination (8, 24). Hence, we inferred 12 HMM states from the concatenated time courses based on minimum free energy and median fractional occupancy, which minimized similarity between different states. More specifically, the HMM analysis captured most of the information contained in the dataset.

Subsequently, we analyzed the dynamic alterations of temporal reconfiguration for all subjects. We found the special temporal characteristics of whole-brain networks related to MELAS-acute patients. In comparison with HCs, we noted that the MELAS cohort manifested increased lifetimes and fractional occupancies in states 8 and 9, along with an elevated switching rate of states. The longer lifetime and higher fractional occupancy of the MELAS-related states in patients demonstrated that these specific network configurations were more sustained and frequently accessed in the MELAS group. These alterations directly reflected the disrupted neural dynamics caused by mitochondrial dysfunction, which impaired the brain’s ability to maintain coherent and efficient communication between distinct network states. The elevated switching frequency further underscored instability, indicating a propensity for uncontrolled transitions between network configurations, which may contribute to the episodic nature of SLEs and the accumulation of neurological dysfunction.

When combining the community detection for transition maps with the global dynamic activity of whole-brain networks, we found that there were special modules of transitions of the HMM states related to MELAS patients and HCs. By using the Neurosynth, MELAS-related modules, primarily featured by states 3 and 8, exhibited significantly decreased activities in crucial areas of the DMN and VN, which was basically consistent with a previous study (6). However, the HC-related module, primarily characterized by states 5 and 6, revealed increased activities in DMN areas, sensorimotor, and visual areas. The identification of distinct community modules highlighted a fundamental difference in how brain networks organize and transition between states in MELAS patients. The MELAS-related module’s emphasis on disrupted DMN and VN dynamics, coupled with their increased presence time and switching frequency, potentially represented the pathological consequence of mitochondrial dysfunction on brain network organization. This altered modular organization likely underpinned the key neuropathological deficits of MELAS, leading to symptoms such as cortical blindness and cognitive impairment associated with VN and DMN dysfunction (25). In addition, we discovered that transitions between states 1, 5, and 6 occurred more often than other states. Notably, states 5 and 6 were part of the HC-associated module, suggesting stronger triangular loops of transitions within the HC group. The stable reconfiguration of transition could be key to understanding the neural network mechanisms. In other words, the occurrence of acute SLE in MELAS patients has disrupted this stable organizational pattern, which could be attributed to insufficient mitochondrial energy production, leading to instability in brain networks. Accordingly, our results provide novel insights about the temporal characteristics and reconfiguration of whole-brain networks in MELAS-acute patients, which may explain the neural network mechanism basis for SLE.

Finally, the positive correlation between elevated LDH levels and longer lifetimes in state 8 provided a direct link between metabolic dysfunction and specific aberrant network dynamics in MELAS. LDH, as an enzyme, plays a key role in the conversion of lactate to pyruvate and is widely distributed in various tissues, which is a marker of cellular stress and impaired energy metabolism. Elevated levels of serum LDH can serve as a significant biomarker in clinical practice, such as mitochondrial dysfunction (26), malignancies (27), and infections (28). This finding solidified state 8 as a distinct “disease-characteristic state,” where prolonged engagement reflected the ongoing metabolic insult to the brain and its resultant impact on network stability and function. Consequently, state 8 and its associated temporal metrics can be considered as potential neuroimaging biomarkers that capture the severity of metabolic impairment and its manifestation in brain network dynamics within MELAS patients.

This study has some limitations. First, the sample size was relatively small, which might impact the statistical power, so the significance of the results could be relatively limited. Future longitudinal cohort studies with larger datasets are needed to better explore the correlations between the temporal characteristics of HMM states and the clinical features of MELAS patients and to potentially establish a prediction model for SLE risk. Second, the number of HMM states is a free parameter, which makes it difficult to ensure an exact number of states. In this study, we aimed to extract as much temporal resolution as possible, though not definitively.

## Conclusion

This study evaluated dynamic alterations in whole-brain networks in MELAS patients using HMM. Our findings revealed a special dynamic reconfiguration of HMM states and transition modules within whole-brain networks, along with multiple transition pathways specific to MELAS. The MELAS-related community was characterized by decreased activities in key areas of the DMN and VN. Moreover, correlation analysis revealed that the concentration of LDH was positively associated with lifetimes in state 8. Therefore, our findings provide a new perspective for elucidating the mechanism of neural network damage in MELAS patients and offer potential biomarkers for evaluating the risk of SLE.

## Data Availability

The raw data supporting the conclusions of this article will be made available by the corresponding authors on reasonable request.

## Footnotes

^1^ http://rfmri.org/dpabi

^2^ https://www.fil.ion.ucl.ac.uk/spm/software/spm12/

^3^ www.graphpad.com

^4^ https://www.mathworks.com/

## Glossary

AAL: Automated anatomical labeling
BOLD: blood oxygen level-dependent imaging
BRAVO: brain volume
CK: creatine kinase
CSF: cerebrospinal fluid
DMN: default mode network
EPI: echo planar imaging
FC: functional connectivity
FD: frame-wise displacement
FO: fractional occupancy
FOV: field of view
HC: healthy control
HMM: hidden Markov model
LDH: lactate dehydrogenase
LT: lifetimes
MELAS: mitochondrial encephalomyopathy with lactic acidosis and stroke-like episodes
MELAS-acute: MELAS patients at acute stage
MNI: Montreal Neurological Institute
ROI: regions of interest
rs-fMRI: resting-state functional magnetic resonance imaging
SF: Switching frequency
SLE: stroke-like episode
SPM: statistical parametric mapping
SWA: sliding windows approach
TE: echo time
TR: repetition time
VN: visual network
WM: white matter.
