# Supplementary material for: Dynamic reconfiguration and transition of whole-brain networks in patients with MELAS revealed by a hidden Markov model
Source: Front Neurol. 2025 Sep 22;16:1625888. doi: 10.3389/fneur.2025.1625888 (PMC12497577; doi:10.3389/fneur.2025.1625888)
Supplement: Supplementary file 1 [file Supplementary_file_1.docx]

**
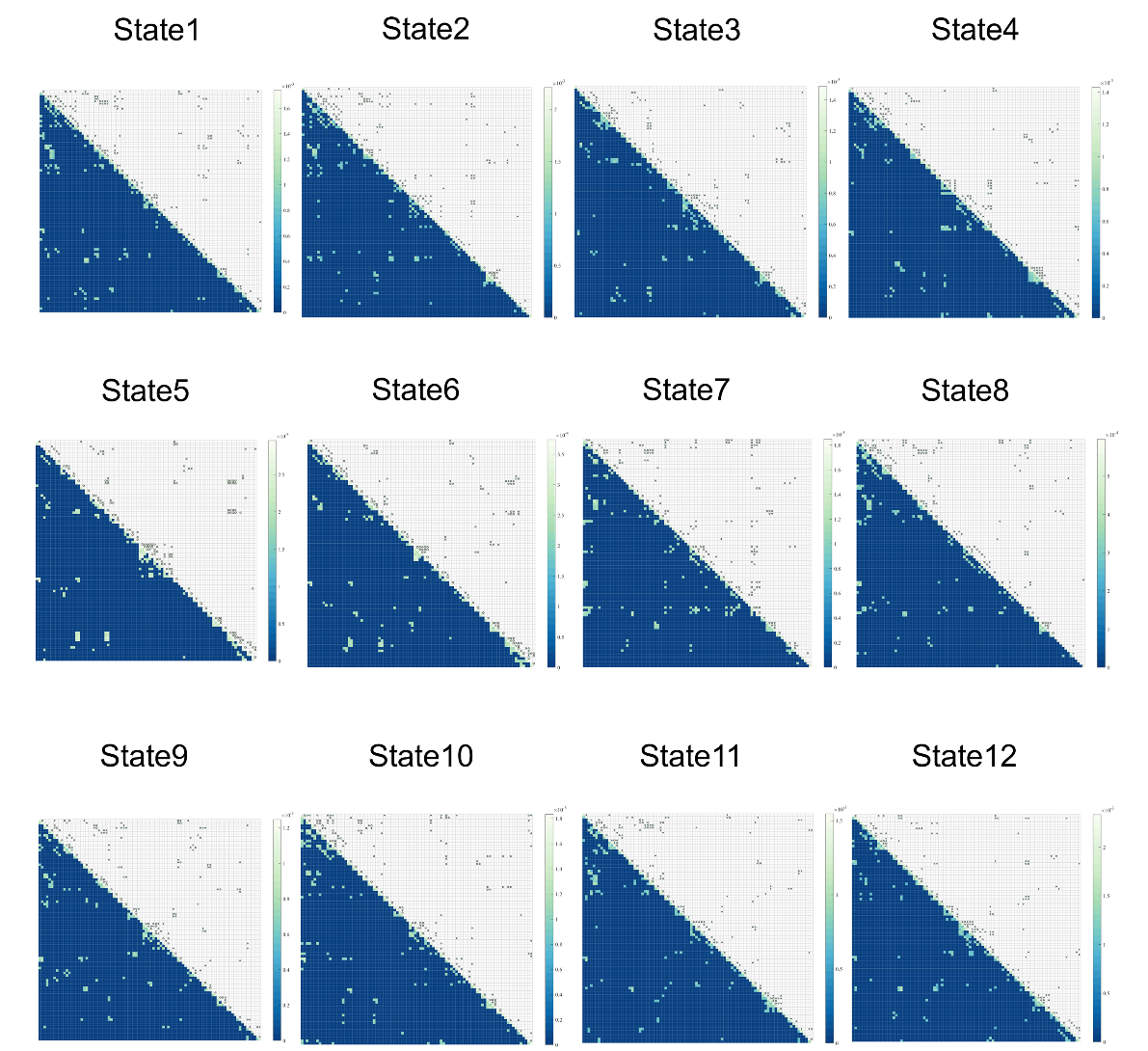
**

**Figure S1** States inferred by HMM. Covariance matrix maps for 12 inferred states.

**
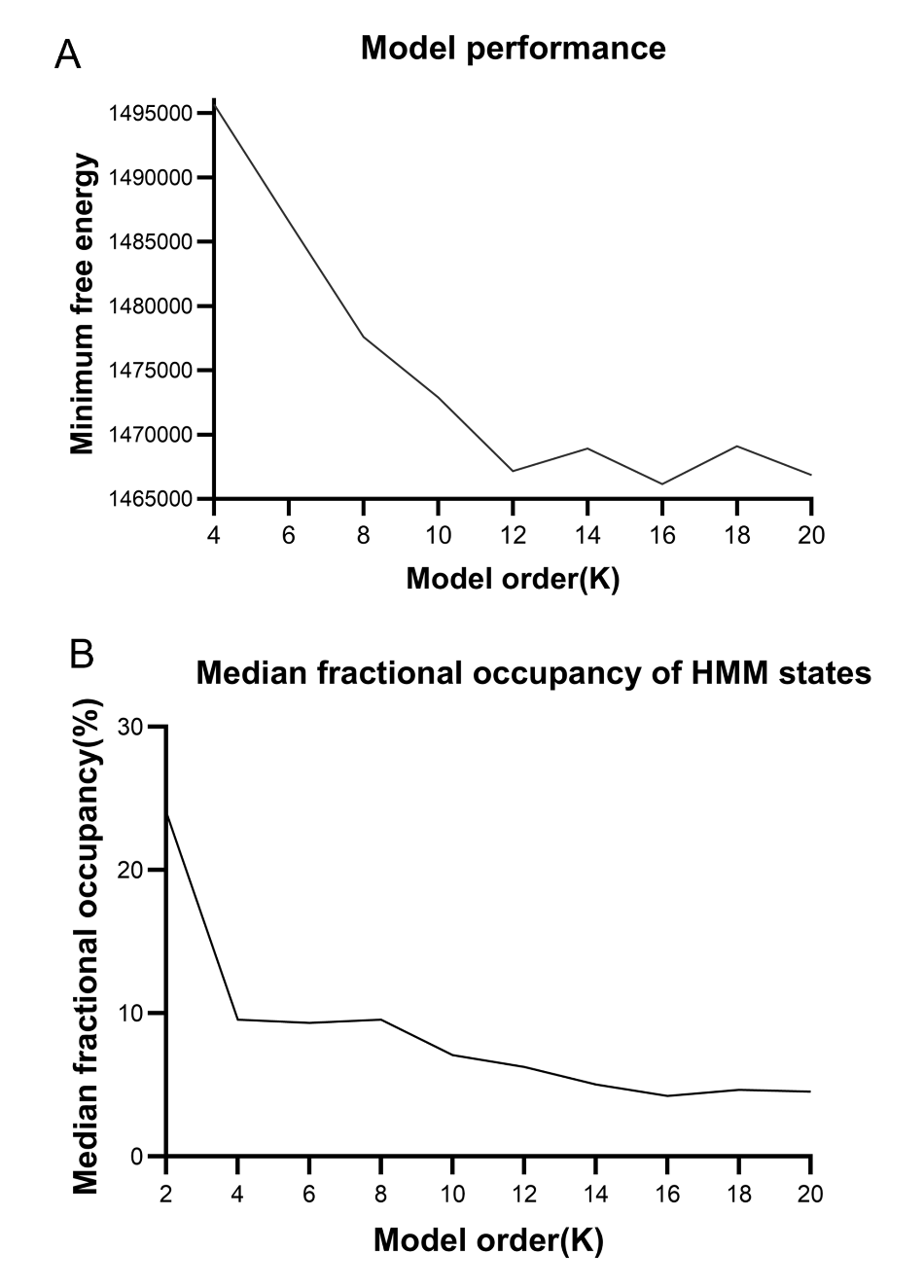
**

**Figure S2** Summary measures of HMM solutions across a range of model orders for the cohort dataset. (A) The minimum free-energy on the inference initially decreased as increasing model orders, while increasing K beyond a certain point resulted in a rise in free energy. (B) The development of the median fractional occupancy as a function

of model order. Note the median fractional occupancy across states remains relatively low and stable without substantial improvement beyond K=12, implying that additional states do not contribute meaningfully to capturing distinct dynamic patterns.
